# Grafting Amino Groups onto Polyimide Films in Flexible Copper-Clad Laminates Using Helicon Plasma

**DOI:** 10.3390/ma16186214

**Published:** 2023-09-14

**Authors:** Chenggang Jin, Chen Wang, Shitao Song, Yongqi Zhang, Jie Wan, Liang He, Ziping Qiao, Peng E

**Affiliations:** 1 Laboratory for Space Environment and Physical Sciences, Harbin Institute of Technology, Harbin 150001, China; cgjin@hit.edu.cn (C.J.); wanjie@hit.edu.cn (J.W.); 2 School of Physics, Harbin Institute of Technology, Harbin 150001, China; 23b311005@stu.hit.edu.cn; 3 School of Electrical Engineering, Liaoning University of Technology, Jinzhou 121001, China; songshitaolnut@163.com; 4 School of Electrical Engineering and Automation, Harbin Institute of Technology, Harbin 150001, China; 21b906046@stu.hit.edu.cn; 5 No. 208 Research Institute of China Ordnance Industries, Beijing 102200, China; hel-152@163.com (L.H.); maryqiao@163.com (Z.Q.)

**Keywords:** flexible copper-clad laminate, polyimide, no adhesive, helicon wave plasma, roughness, peel strength

## Abstract

Polyimide (PI) films are widely used in electronic devices owing to their excellent mechanical and electrical properties and high thermal and chemical stabilities. In particular, PI films play an important role in flexible printed circuit boards (FPCBs). However, one challenge currently faced with their use is that the adhesives used in FPCBs cause a high dielectric loss in high-frequency applications. Therefore, it is envisioned that PI films with a low dielectric loss and Cu films can be used to prepare two-layer flexible copper-clad laminates (FCCLs) without any adhesive. However, the preparation of ultra-thin FCCLs with no adhesives is difficult owing to the low peel strength between PI films and Cu films. To address this technical challenge, an FCCL with no adhesive was prepared via high-power helicon wave plasma (HWP) treatment. Field-emission scanning electron microscopy (FE-SEM), X-ray photoelectron spectroscopy (XPS), and X-ray diffraction (XRD) were tested. Also, the surface roughness of the PI film and the peel strength between the PI film and Cu film were measured. The experimental results show that the surface roughness of the PI film increased by 40–65% and the PI film demonstrated improved adhesion (the peel strength was >8.0 N/cm) with the Cu film following plasma treatment and Cu plating.

## 1. Introduction

In recent years, with the development of small and light electronic devices, the demand for flexible electronic components has increased. In particular, flexible printed circuit boards (FPCBs) have garnered great interest. An FPCB with excellent material performance needs to be prepared with high-quality, flexible copper-clad laminates (FCCLs). Owing to their excellent mechanical and electrical properties [1] as well as high chemical and thermal stabilities [2], polyimide (PI) films have become an important material for preparing FCCLs [3,4,5,6,7]. However, owing to their low roughness and smooth surface, PI films have a low adhesion strength with Cu films [8], necessitating the use of adhesives. Further, many adhesives exhibit high dielectric loss during high-frequency applications. Therefore, to manufacture adhesive-free FPCBs, PI films need to be modified to increase their adhesion strength with Cu films prior to FCCL preparation [9,10,11].

Conventional PI film surface modification methods include acid or alkali treatment [12,13,14], ion beam etching [15], and grafting functional groups onto a surface via plasma treatment [16,17,18,19]. Acid or alkali treatment is a surface modification method used to increase the surface energy and adhesive properties of PI films by hydrolyzing the surface imide group into polyamide acid and its associated metal salt. The peel strength of PI films after acid or alkali treatment has been shown to be >7.9 N/cm [14]. Since PI does not exhibit high alkaline resistance, alkaline hydrolysis can easily and efficiently change the structure and morphology of the PI film surface. Likewise, ion beam etching improves the mechanical and adhesive properties of PI films by controlling molecular aggregation through physical methods. The peel strength of PI films after ion beam etching has been shown to be >7.0 N/cm [15]. The main parameters that control the effectiveness of ion beam etching are the energy, ion current density, and etching time. Finally, grafting functional groups onto a surface via plasma treatment is a method that has become increasingly popular in recent years. Non-thermal plasma treatment in particular is often used for the surface modification of polymeric materials. The peel strength of the PI films after grafting functional groups onto a surface via plasma treatment has been shown to be >8.0 N/cm [17]. Plasma is considered a fully or partially ionized gas state of a substance [20], which may contain atoms, molecules, metastable ions, and excited ions. The electron, anion, and cation contents in the plasma are nearly the same. Plasma has a high energy density and easily undergoes physical and chemical reactions with other substances. Grafting amino groups onto PI films with plasma can quickly change the surface composition of PI films without affecting their bulk-phase composition. This method can be optimized by identifying suitable processing parameters, thereby creating the ideal working conditions for subsequent treatment following grafting of the amino groups.

Herein, amino groups were grafted onto a PI film using helicon wave plasma (HWP) for the first time. An HWP source is a low-temperature plasma source which has a high plasma density, high ionization efficiency [21], high and uniform electron density, low confining magnetic field, strong controllability [22], and a relatively simple structure. The HWP source can heat a large area of electrons when the helicon wave propagates in the plasma and can discharge at an ultra-low pressure. Helical antennas play an important role in providing the radio frequency power needed for ionizing and heating plasma in the HWP discharge. Based on the results of numerical simulations and experimental research on plasma excited by a helicon wave [23,24,25], a right helical antenna of a half wavelength was adopted. In this work, an ultra-high radio frequency power of up to 10 kW was used, leading to an increase in the electron density. The surface roughness and peel strength of the PI film notably increased after the PI film surface was grafted with amino groups using high-power HWP. The surface roughness of the PI film increased by 40–65% following the plasma treatment, and the PI film demonstrated improved adhesion strength (the peel strength was >8.0 N/cm) with the Cu film after plasma treatment and Cu plating.

## 2. Experimental Section

### 2.1. Material Preparation

The PI films (HN type, 50 mm × 50 mm, 25 μm thick, Du Pont, Wilmington, DE, USA) were purchased from a local vendor and were cut into 50 mm × 50 mm pieces, which served as the substrates for surface metallization. After rinsing them with deionized water several times, the PI films were degreased in an ultrasonic bath containing ethanol solution for 30 min, and then dried in an oven at 60 °C for 30 min. The as-cleaned PI films were placed in the chamber of a plasma system subjected to a CH_4_ (99.999%) and NH_3_ (99.999%) flow of 200 standard cubic centimeters per min (sccm). The working pressure of the plasma treatment was maintained at 0.5 Pa. The radio frequency power with a frequency of 13.56 MHz was set to 4 kW to generate the NH_3_/CH_4_ plasma used to cover the PI surface for several minutes. The PI substrates had a pulsed bias voltage (V_s_) within a range of −400–0 V with a frequency of 10 kHz, duty ratio of 20%, and sample temperature of 30 °C, which was measured using a thermocouple in real time. After the plasma process, the samples were cooled to approximately 25 °C under N_2_ flow. Subsequently, the specimens were sectioned with a width of 1 mm, and used to probe the surface properties.

Cu films were prepared using self-designed ultra-high-vacuum multi-functional sputtering equipment manufactured by SKY Technology Development Corporation Limited, Chinese Academy of Sciences, Shenyang, China. The system was pumped, achieving a base pressure of 1 × 10^−5^ Pa before Cu plating. The Cu target (3 inch) was purchased from GRINM Group Corporation Limited, Beijing, China. In the Cu plating process, the radio frequency sputtering power ranged from 50 to 120 W, the duration of sputtering was 10 min, and 99.9999% pure Ar was selected as the working gas. There was no additional heating during Cu plating. Unless otherwise specified, the thickness of Cu films was 5 μm.

Optical emission spectroscopy (OES) profiles were collected using a grating spectrometer (Princeton Instruments, Trenton, NJ, USA, HRS-750S) and an intensified charge-coupled device (ICCD) camera (Princeton Instruments, PM4-1024i), in combination with a 74-UV silica-collimating lens. The spectra were measured for wavelengths between 200 and 800 nm in increments of 0.03 nm.

### 2.2. Morphological and Structural Characterization

Field-emission scanning electron microscopy (FE-SEM, SU8010, Hitachi, Tokyo, Japan) was employed to observe the morphology and thickness of the coating. X-ray photoelectron spectroscopy (XPS) was used to analyze the elemental composition and chemical bonding of the substrate. An ESCALAB 250XI spectrometer was used in combination with in situ Ar^+^ ion etching and equipped with a monochromatic Al K_α_ (hυ = 1486.6 eV) X-ray source. The base pressure during spectral acquisition was at least 6.6 × 10^−10^ mbar. The analyzed sample area was 650 μm. The electron emission angle was set to 58°. The sample sputter-etched period was 10 min, the Ar^+^ energy was 1000 eV, and the incidence angle was set to 40° from the vertical axis. Charge neutralization was used during the XPS experiment. The charge-referencing method used was the work function method. X-ray diffraction (XRD) analysis was performed using an X-ray diffractometer (Bruker D8 Advance, Mannheim, Germany), with a Cu K_α_ line at 0.15418 nm as a source, in the range of 10–80° at a scanning rate of 5°/min.

### 2.3. Mechanical Property Characterization

The roughness was calculated from the roughness profile determined via atomic force microscopy (AFM). The roughness data are shown in Table 1. Peeling tests were performed to measure the force required to peel off the film per unit width, and the strength is reported in N/cm. The peeling test protocol was followed as described in the literature [26]. A 90° peel test was performed in this study. The peeling test between the Cu films and PI films was performed six times, and the average was reported as the peel strength. The peel strength data are shown in Table 2, Table 3 and Table 4.

## 3. Results and Discussion

**Figure 1:**
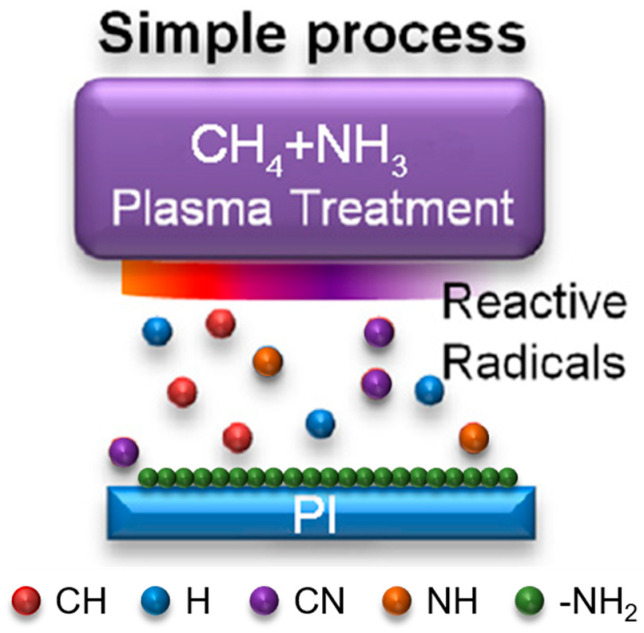
Schematic depicting the grafting of amino groups onto PI films through HWP treatment.

**Figure 2:**
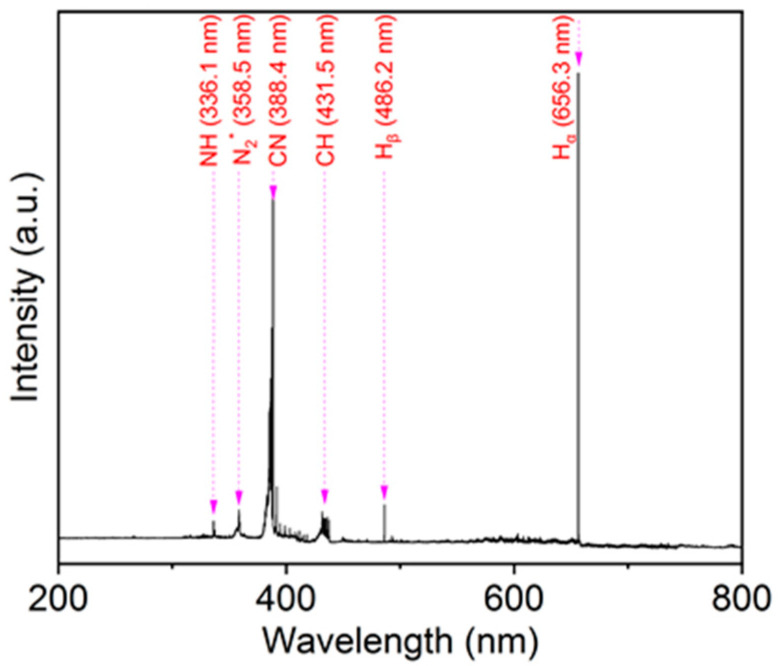
OES profiles of the CH_4_/NH_3_ HWP discharge in the wavelength range of 200~800 nm.

HWP was used to graft amino groups onto the surface of PI films. The HWP device consists of a plasma source and a material processing chamber. CH_4_/NH_3_ was decomposed into reactive radicals in the chamber (Figure 1). During the HWP discharge, OES was used to study the correlation between the grafted amino groups and various other active species. Since the applied V_s_ has no effect on OES, the typical emission spectrum during HWP discharge at a −V_s_ of 100 V is displayed in Figure 2 for the spectral range of 200–800 nm. Intense emission peaks of reactive species were observed in the plasma discharge. Specifically, the emission peaks of NH (336.1 nm), N_2_* (358.5 nm), CN (388.4 nm), CH (431.5 nm), H_β_ (486.2 nm), and H_α_ (656.3 nm) were observed. The N_2_* molecular spectra likely arose from the surface air discharge between the outer surface of the reactor and the inner surface of the grounding electrode. According to the OES results in the literature [27], a possible fragmentation pathway is as follows: CH_4_ + NH_3_ + e^−^→NH, CN, CH, H. This pathway indicates that a higher *n_e_* can enhance or promote the dissociation of the precursor. NH and CN transitions were observed in the OES profiles, arising from the decomposition of CH_4_/NH_3_ during the HWP discharge, which can be considered as a reaction precursor for the grafting of amino groups. Many energetic H atoms are generated from CH_4_/NH_3_ by the HWP discharge, and these H atoms have the ability to eliminate certain unstable phases.

**Figure 3:**
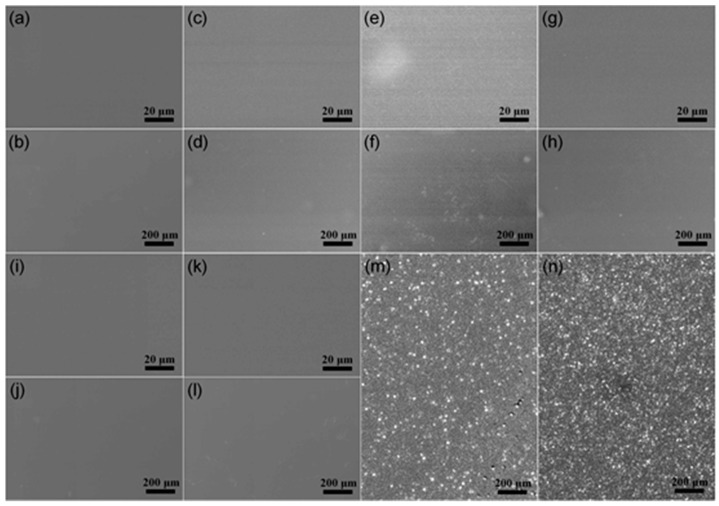
FE-SEM images of PI films under no treatment and plasma treatment at various V_s_ values: (**a**,**b**) no treatment; (**c**,**d**) 0 V; (**e**,**f**) −100 V; (**g**,**h**) −200 V; (**i**,**j**) −300 V; (**k**,**l**) −400 V. FE-SEM images of (**m**) a PI film under no treatment after Cu plating and (**n**) a PI film subjected to plasma treatment at a −V_s_ of 100 V after Cu plating.

The morphology of the PI films was analyzed using FE-SEM after the amino groups were grafted onto the PI films via high-power HWP. As illustrated in Figure 3a–l, the surface morphology of the PI films changed following plasma treatment. Notably, the surface morphology changes were most pronounced at the −V_s_ of 100 V, where the roughness of almost the entire surface increased. This result indicates that the effect of plasma treatment is optimal at a −V_s_ of 100 V. Next, the morphologies of the plasma-treated PI film at −V_s_ = 100 V and the non-treated PI film were compared following Cu plating. As shown in Figure 3m,n, the Cu clusters on the surface of the PI film were not compact after Cu plating on the PI film without plasma treatment and no films were formed, indicating that it is difficult to plate Cu on PI films without plasma treatment. Further, the Cu atoms on the surface of the PI film formed compact clusters after Cu plating on the PI film with plasma treatment at a −V_s_ of 100 V, and a compact film was formed, indicating that the effect of Cu plating on PI films with plasma treatment at a −V_s_ of 100 V is very good. Therefore, it can be concluded that plasma treatment can promote Cu plating on PI films. However, the mechanism behind why plasma treatment enhances the Cu plating ability is still unknown and necessitates further investigation and discussion.

**Figure 4:**
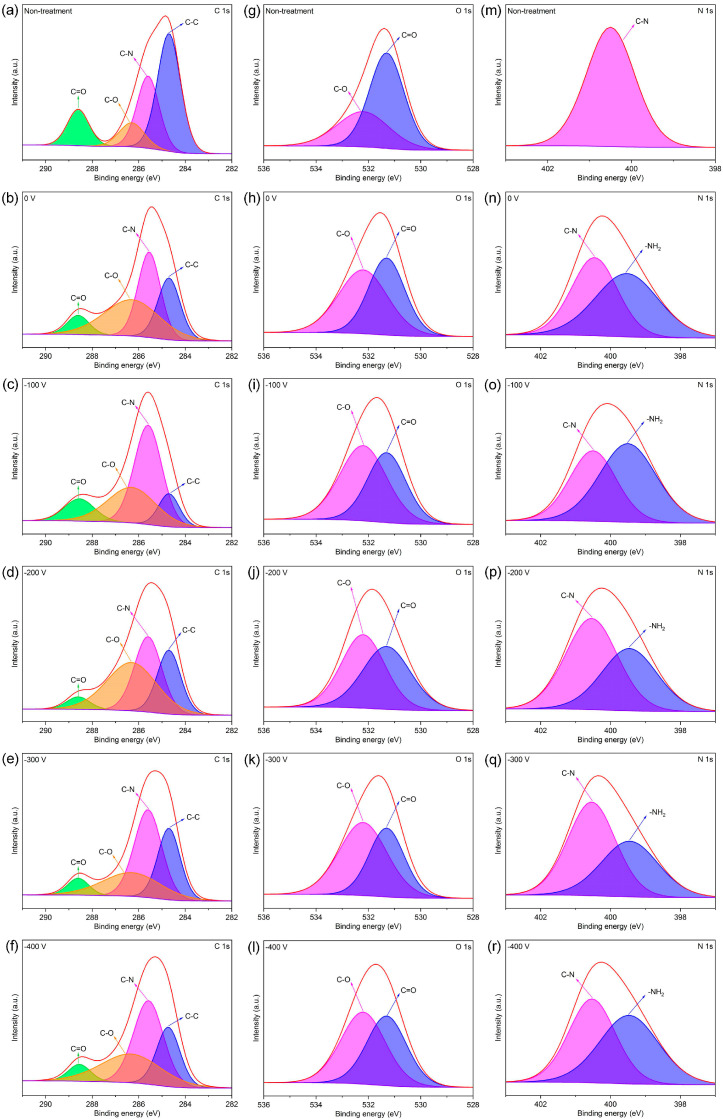
C 1s, O 1s, and N 1s XPS profiles of the PI films after no treatment and plasma treatment at various V_s_ values: (**a**) C 1s, no treatment; (**b**) C 1s, 0 V; (**c**) C 1s, −100 V; (**d**) C 1s, −200 V; (**e**) C 1s, −300 V; (**f**) C 1s, −400 V; (**g**) O 1s, no treatment; (**h**) O 1s, 0 V; (**i**) O 1s, −100 V; (**j**) O 1s, −200 V; (**k**) O 1s, −300 V; (**l**) O 1s, −400 V; (**m**) N 1s, no treatment; (**n**) N 1s, 0 V; (**o**) N 1s, −100 V; (**p**) N 1s, −200 V; (**q**) N 1s, −300 V; (**r**) N 1s, −400 V.

To investigate whether the amino groups were successfully grafted onto the PI film surface, the chemical bonds of the PI films following amino group grafting were analyzed by measuring the XPS profiles of the plasma-treated PI film. As shown in Figure 4a–f, the peaks at approximately 288.6, 286.3, 285.6, and 284.7 eV are the characteristic peaks of the C=O [28], C−O [29,30], C−N [31], and C−C bonds [32], respectively. The intensity of the characteristic peak of the C−N bond is the highest at the −V_s_ of 100 V. As shown in Figure 4g–l, the peaks at approximately 532.2 and 531.3 eV are the characteristic peaks of the C−O and C=O bonds [33], respectively. In Figure 4m–r, the spectra of the plasma-treated PI films exhibit the characteristic peaks of the −NH_2_ [34] and C−N bonds [35,36], where the binding energies of the N 1s electrons are approximately 399.5 and 400.5 eV, respectively. However, the spectrum of the untreated PI film only contains the characteristic peak of the C−N bond, where the binding energy of the N 1s electrons is approximately 400.5 eV. The characteristic peak intensity of the −NH_2_ group is the highest at the −V_s_ of 100 V. The above results indicate that the amino groups were successfully grafted onto the surface of the plasma-treated PI films and that the degree of amino group grafting was optimized at a −V_s_ of 100 V.

**Figure 5:**
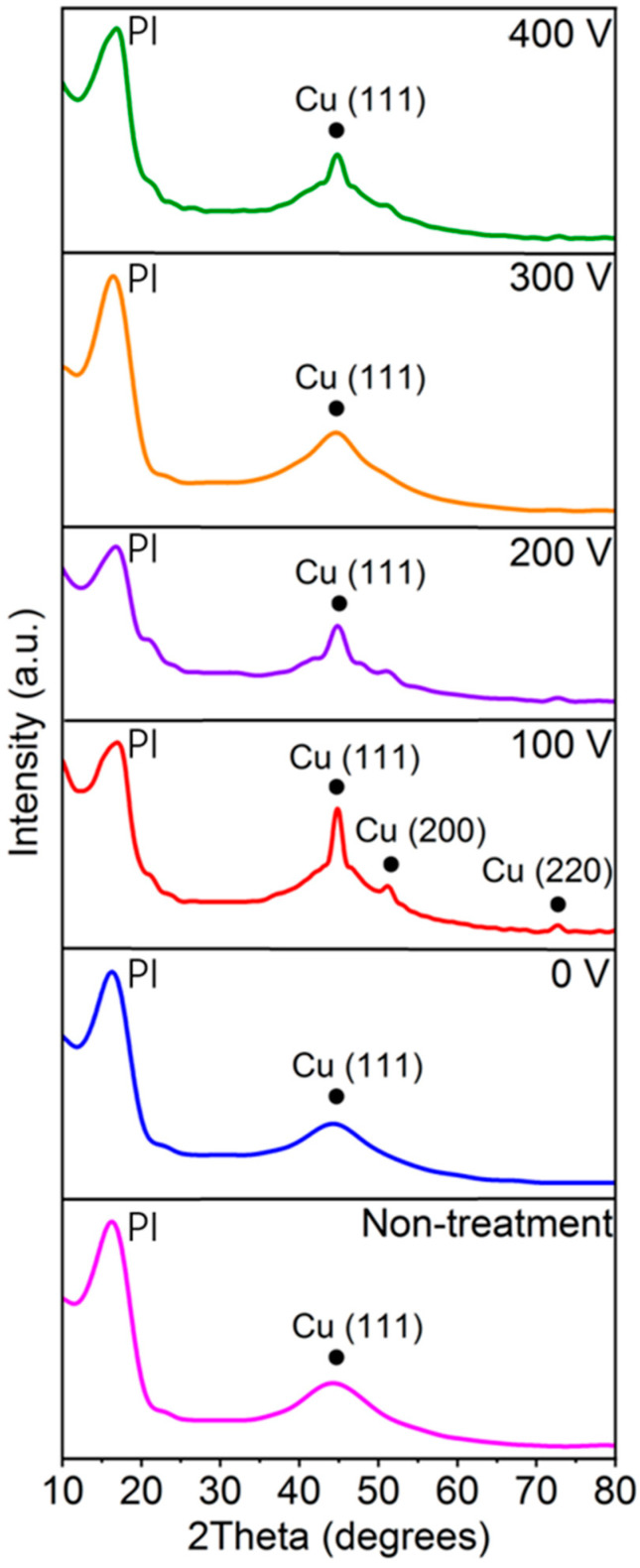
XRD patterns of Cu-plated PI films with plasma treatment at various −V_s_ values and without plasma treatment.

To further characterize the structure of the PI films after Cu plating, the XRD patterns of the treated and non-treated PI films post Cu plating were acquired, and the results are shown in Figure 5. The peak at approximately 16.5° is the characteristic peak of PI [37], and the peaks at approximately 44°, 51°, and 73° are the characteristic peaks of the Cu (111), Cu (200), and Cu (220) crystal planes [38,39], respectively. As shown in Figure 5, the characteristic peak intensity of Cu was very low after Cu plating on the non-treated PI film, indicating that it is difficult to plate Cu on non-treated PI films. With an increasing −V_s_ value, the effectiveness of grafting amino groups becomes better and better. Among all the PI films plasma-treated at various V_s_ values, the characteristic peak intensity of Cu was the highest after Cu plating on the PI film with plasma treatment at −V_s_ = 100 V, indicating that this plasma-treated PI film closely bonded with the Cu film, since amino group grafting is optimal at these parameters. When the −V_s_ value is more than 100 V, the effectiveness of the treatment starts to randomly decline, at which point the effectiveness of Cu plating also starts to randomly decline.

**Figure 6:**
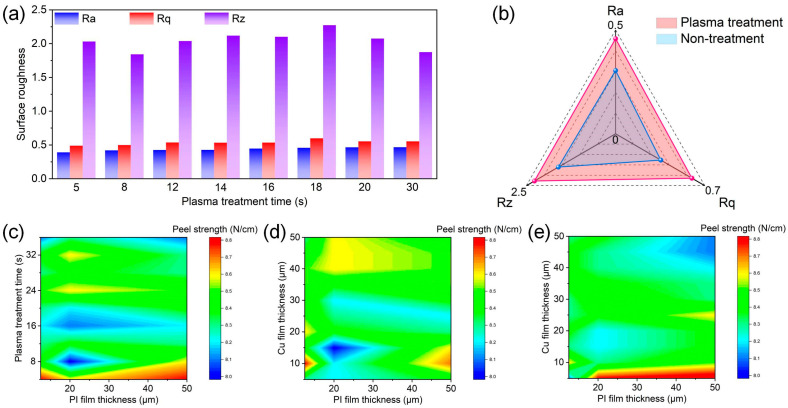
(**a**) Surface roughness of the plasma-treated PI films at various times; (**b**) surface roughness of PI films after plasma treatment and no treatment; (**c**) peel strength between Cu films and PI films with various thicknesses after plasma treatment for various times and sputtering Cu plating; (**d**) peel strength between Cu films with various thicknesses and PI films with various thicknesses after plasma treatment for 5 s and sputtering Cu plating; (**e**) peel strength between Cu films with various thicknesses and PI films with various thicknesses after plasma treatment for 5 s and electroless Cu plating.

To investigate whether the plasma-treated PI films could improve Cu film adhesion, the surface roughness of the PI films was measured. The surface roughness can reflect the degree of adhesion caused by uneven surfaces of PI films when Cu films are attached to PI films. PI films with rougher surfaces have larger surface areas, and therefore, exhibit stronger adhesion. Figure 6a displays the surface roughness of the plasma-treated PI films at various times. As shown in Figure 6b, when compared to the untreated PI film, the surface roughness of the PI film increased by 40–65% following plasma treatment. The increase in the surface roughness of the PI film was very beneficial for increasing the adhesion after sputtering/electroless Cu plating [40].

To further investigate whether the PI films subjected to plasma treatment could closely bond with the Cu films, the peel strength between the PI films and Cu films was measured. In the case of pure elastic, the peeling force F (N/cm) corresponds to the energy required to separate the Cu/PI interface [26]. The peel strength between the PI films and Cu films also reflects the degree of adhesion between the PI films with Cu films; a higher peeling strength indicates closer bonding. As shown in Figure 6c, the PI film had good adhesion (the peel strength is >8.0 N/cm) with the Cu film following plasma treatment and Cu plating, and the thickness of the PI films did not affect its adhesion abilities. With the increase in plasma treatment time, the effectiveness of grafting amino groups became better and better. When the plasma treatment time was 5 s, the effectiveness of grafting amino groups reached the maximum value, at which point the peel strength reached the maximum value. When the plasma treatment time was longer than 8 s, the effectiveness of the treatment was still satisfactory, but subsequently started to decline, at which point the peel strength also started to decline. Therefore, the optimal plasma treatment time is between 5 and 8 s. Figure 6d shows the peel strength between the Cu films and PI films, both with various thicknesses, following plasma treatment for 5 s and sputtering Cu plating. The results demonstrate that sputtering Cu plating is suitable for preparing FCCLs with thicker Cu films. This is because the film–substrate bonding strength of sputtering Cu plating is strong. The peel strength will not decrease with an increase in Cu film thickness. Figure 6e depicts the peel strength between the Cu films and PI films, both with various thicknesses, following plasma treatment for 5 s and electroless Cu plating. The results show that the quality of FCCLs prepared via electroless Cu plating is comparable to that of the FCCLs prepared via sputtering, but electroless Cu plating is more suitable for preparing FCCLs with thinner Cu films. This is because the film–substrate bonding strength of electroless Cu plating is weak. With the increase in Cu film thickness, the peel strength will decrease.

## 4. Conclusions

A high-power HWP device for surface treatment was designed and fabricated, and amino groups were grafted onto a PI film surface using this device. The FE-SEM and XPS results reveal that the amino groups were successfully grafted onto the PI film. Amino grafting was optimal at a −V_s_ of 100 V. Additionally, the XRD results show that the PI film subjected to lamination treatment adhered well to the Cu film at a −V_s_ of 100 V. In addition, the surface roughness of the PI film and its peel strength with Cu film following the grafting of the amino groups were measured. The experimental results show that the surface roughness of the PI film increased by 40–65% following plasma treatment and that the PI film adhered well (the peel strength was >8.0 N/cm) with the Cu film after plasma treatment and Cu plating. In other words, the surface roughness of the PI film subjected to plasma treatment noticeably increased, which was very beneficial for improving its adhesion after sputtering/electroless Cu plating. These results demonstrate that it is possible to prepare FCCLs without the use of adhesives, including ultra-thin FCCLs with a high peel strength. Moreover, the preparation method outlined in this paper is relatively simple, which can reduce environmental waste, reduce the amount of human resources needed, and reduce manufacturing costs.

## Figures and Tables

**Table 1:** The surface roughness of PI films.

Plasma Treatment Time (s)	Plasma Treatment			No Treatment		
	Ra	Rq	Rz	Ra	Rq	Rz
5	0.391	0.489	2.031	0.312	0.363	1.353
8	0.42	0.498	1.842	0.318	0.37	1.321
12	0.426	0.537	2.039	0.295	0.344	1.515
14	0.427	0.534	2.119	0.257	0.297	1.147
16	0.447	0.535	2.101	0.263	0.329	1.408
18	0.458	0.599	2.273	0.305	0.355	1.604
20	0.465	0.554	2.075	0.282	0.348	1.335
30	0.467	0.553	1.875	0.271	0.326	1.175

**Table 2:** Peel strength between Cu films and PI films with various thicknesses after plasma treatment for various times and sputtering Cu plating.

Plasma Treatment Time (s)	0	4	8	12	16	20	24	28	32	36	PI Film Thickness (μm)
Peel strength (N/cm)	0	8.8	8.4	8.3	8.2	8.3	8.5	8.3	8.4	8.1	12.5
	0	8.6	8.0	8.4	8.1	8.2	8.6	8.4	8.6	8.3	20
	0	8.8	8.6	8.3	8.2	8.4	8.5	8.3	8.3	8.1	50

**Table 3:** Peel strength between Cu films with various thicknesses and PI films with various thicknesses after plasma treatment for 5 s and sputtering Cu plating.

Cu Film Thickness (μm)	5	10	15	20	25	30	40	50	PI Film Thickness (μm)
Peel strength (N/cm)	8.3	8.8	8.4	8.6	8.5	8.5	8.5	8.3	12.5
	8.2	8.2	8.0	8.4	8.3	8.2	8.6	8.6	20
	8.4	8.7	8.6	8.3	8.2	8.4	8.5	8.4	50

**Table 4:** Peel strength between Cu films with various thicknesses and PI films with various thicknesses after plasma treatment for 5 s and electroless Cu plating.

Cu Film Thickness (μm)	5	10	15	20	25	30	40	50	PI Film Thickness (μm)
Peel strength (N/cm)	8.1	8.6	8.3	8.4	8.3	8.3	8.4	8.3	12.5
	8.8	8.3	8.2	8.2	8.4	8.5	8.3	8.4	20
	8.9	8.5	8.4	8.4	8.6	8.3	8.2	8.1	50

## Data Availability

Data will be made available on request.
